# Observations of the Effects of Maternal Fasting Plasma Glucose Changes in Early Pregnancy on Fetal Growth Profiles and Birth Outcomes

**DOI:** 10.3389/fendo.2021.666194

**Published:** 2021-08-19

**Authors:** Fei Guo, Yindi Liu, Zheng Ding, Yong Zhang, Chen Zhang, Jianxia Fan

**Affiliations:** ^1^Department of Obstetrics and Gynecology, The International Peace Maternity and Child Health Hospital, School of Medicine, Shanghai Jiao Tong University, Shanghai, China; ^2^Shanghai Key Laboratory of Embryo Original Diseases Affiliated to Shanghai Jiao Tong University School of Medicine, Shanghai, China; ^3^Shanghai Key Laboratory of Embryo Original Disease, Shanghai, China

**Keywords:** pregnancy, fetal grow, modification effects, body mass index****, fasting plasma glucose

## Abstract

**Introduction:**

Although the role of maternal hyperglycemia on birth outcomes is clear, literature regarding fetal growth is scarce. We examined the possible associations between maternal fasting plasma glucose (FPG) and fetal growth.

**Materials and Methods:**

A total of 35,981 singleton-pregnant women with FPG in the first trimester were included. Fetal growth parameters were measured during pregnancy by ultrasound at mid and late pregnancy. Information on birth characteristics was retrieved from medical records. We used multivariable linear and logistic regression to determine the associations between FPG and z-scores of fetal parameters and risks of birth outcomes and to assess effect modification by maternal characteristics.

**Results:**

A per-unit increase in FPG levels was negatively associated with fetal parameters in mid pregnancy but positively correlated with those in late pregnancy and with birth characteristics. The effect estimates in late pregnancy were attenuated by maternal pre-pregnancy body mass index (BMI). A significant relationship between FPG and abdominal circumference (AC), an indicator of fetal adiposity, was sustained in subgroups of women with advanced age, positive family history of diabetes, and multiparity in fully adjusted models. After stratification by BMI, high FPG was associated with accelerated AC only in normal controls (0.044 SD; 95% CI: 0.010, 0.079) and overweight/obese women (0.069 SD; 95% CI: -0.002, 0.140) but not in underweight women. High FPG was an independent risk factor for large-for-gestational age in the whole group and stratified subgroups.

**Conclusions:**

Increased FPG in early pregnancy is closely related to fetal growth. Maternal characteristics may modify the associations between FPG and fetal adiposity in late pregnancy.

## Introduction

Since the human fetus is highly dependent on glucose derived from maternal circulation, the glucose homoeostasis transferred from mother to placenta is considered to be the dominant determinant of fetal development (1). Substantial studies have shown that a higher gestational glycemia in each trimester, regardless of fasting or postprandial state, is associated with increased risks of adverse birth outcomes, even in non-diabetic pregnancy (2–5). However, the majority of neonates with abnormal fetal growth are unidentified until birth. Given the prolonged exposure to hyperglycemia from early pregnancy, the impact of metabolic variation in mothers on the fetus in uterus is poorly understood. This notion is reinforced by the view that birth weight is only the endpoint of different fetal exposures, and different fetal growth parameters and body proportions may result in the same birth size (6). Altered fetal growth is a critical predictor of neonatal morbidity and mortality and may increase the susceptibility to multiple diseases later in life (7). For instance, accelerated fetal growth predisposes individuals to obesity later in life, and fetal growth retardation is related to adult diabetes and cardiovascular diseases (8–10).

To our knowledge, only two studies have reported the impact of maternal blood glucose in early pregnancy on fetal growth trajectories. Specifically, Geurtsen et al. (11) reported that high maternal early-pregnancy random blood glucose levels contributed to decreased fetal growth in mid pregnancy and increased fetal growth from late pregnancy onward in the US. Li et al. (12) only found early-pregnancy random blood glucose increased fetal growth in late pregnancy in China. However, non-fasting glucose levels can be affected by the collection date and timing of the last meal and may not truly represent women’s insulin resistance levels. At present, there are few reports about the effect of maternal fasting plasma glucose (FPG) levels in early pregnancy, which are relatively steady, on fetal intrauterine growth. In addition, the International Association of Diabetes and PregnanThe associations with LGAcy Study Groups (IADSPG) has once recommended women with FPG of ≥5.1 mmol/L as having early gestational diabetes mellitus (GDM) (13). However, little is known about fetal growth trajectories in early GDM and non-early GDM pregnancies. Meanwhile, easily obtained clinical subject characteristics such as family history of diabetes, maternal age, parity, fetal sex, and pre-pregnancy BMI, are important factors influencing intrauterine growth and birth outcomes, but few studies have investigated the potential interplay between circulating glucose and these variables (14–18).

In this study, our primary objective was to quantify the associations between maternal first-trimester FPG and the processes of fetal growth in different developmental periods. Our secondary objective was to explore potential modifiers.

## Materials and Methods

### Study Participants

**Figure 1:**
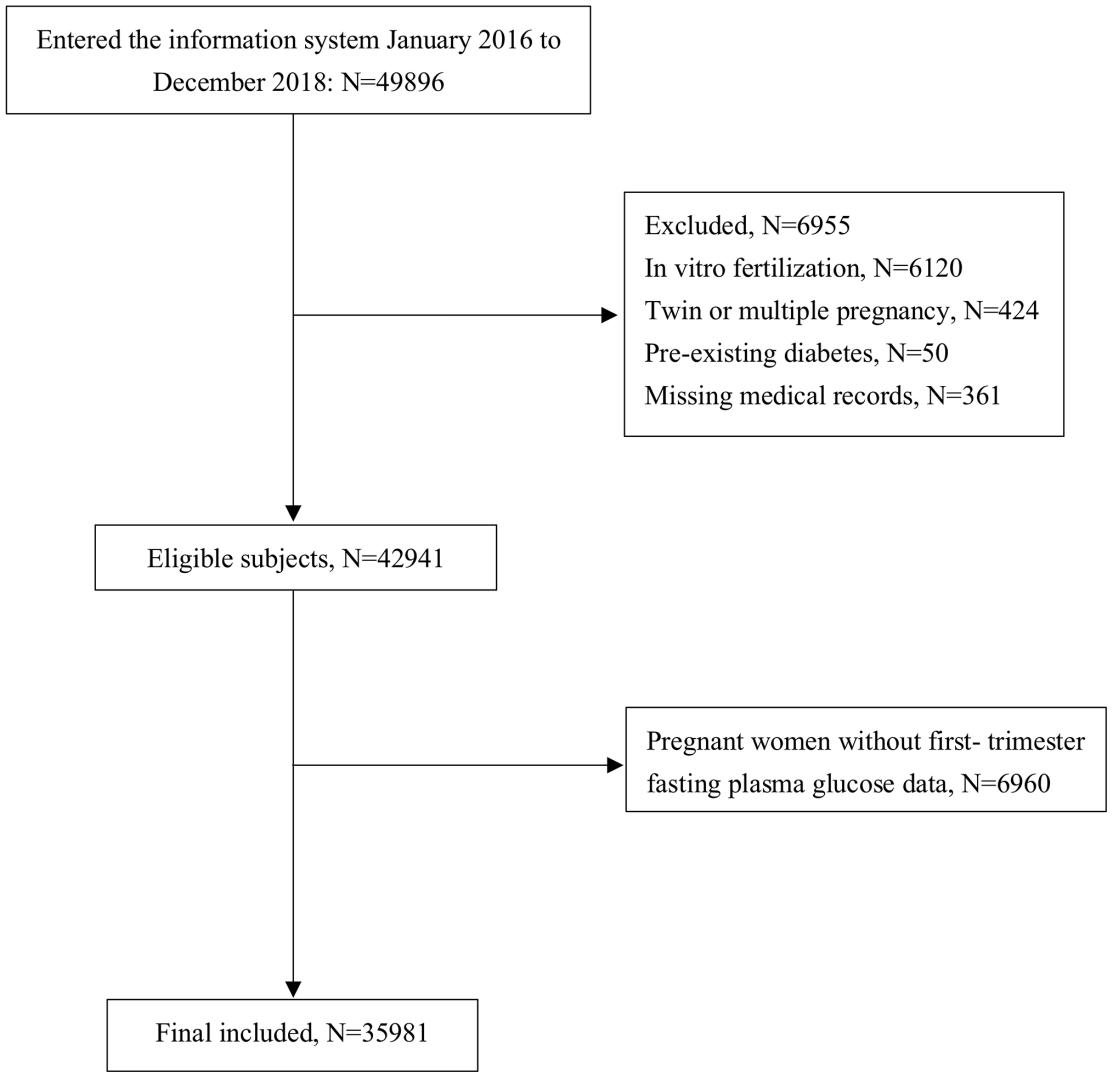
Flowchart of the study population.

Retrospective medical records of pregnant women who underwent their first trimester antenatal care in the International Peace Maternity and Child Health Hospital (IPMCH) in Shanghai, China, from January 2016 to December 2018, were obtained. Women who did not take the FPG test in the first trimester (9–14 weeks), had twin or multiple pregnancies, had preexisting diabetes, became pregnant through *in vitro* fertilization treatment or the use of ovulation stimulation drugs, or had incomplete medical data were excluded from the analysis. A total of 35,981 women were included in our study after excluding the abovementioned subjects (**Figure 1**). To evaluate potential selection bias, we conducted non-response analyses to compare the characteristics between the included (N = 35,981) and excluded pregnant women without first-trimester FPG data (N = 6,960). The results showed that there was no significant difference in maternal age, pre-pregnancy BMI, or birth outcomes, except for that mothers with FPG measurements had a higher proportion of multiparity (69.22% *vs*. 61.26%, p = 0.001), a lower rate of preterm birth (5.25% *vs*. 5.86%, p = 0.039), and slightly higher gestational age (39.1 *vs*. 39 weeks, p < 0.001) (**Supplementary Table 1**). The study was approved by the ethics committee of IPMCH (GKLW 2019-58) and performed in accordance with the Declaration of Helsinki. Written informed consent was obtained from all participants.

### Data Collection

During the first antenatal visit, fasting blood samples were drawn for later measurement of glucose. Information about maternal age, education level, obstetrical history, last menstrual period (LMP), family history of diabetes, and anthropometry measurements were extracted from the medical record system. Previous study reported that advanced maternal age, defined as 35 years or greater, plays an important role in fetal growth (19). Therefore, maternal age was categorized as young (<35 years) and advanced age (≥35 years) in this study. Pre-pregnancy body mass index (BMI; kg/m^2^) was calculated using nurse-measured height in early pregnancy and self-reported weight from weight prior to pregnancy. Pre-pregnancy BMI was calculated as weight divided by height squared and was further classified into three groups: underweight (<18.5 kg/m^2^), normal weight (18.5–23.9 kg/m^2^), and overweight/obese (≥24.0 kg/m^2^). Dichotomous variables were used to indicate maternal parity (nulliparous and multiparous), fetal sex (male and female), and family history of diabetes (positive and negative). Gestational age was estimated using ultrasound screening of the crown–rump length (CRL). If the difference between LMP and CRL based on gestational age was >10 days, we chose the latter method.

Women who have not previously been diagnosed with diabetes underwent a 75-g oral glucose tolerance test (OGTT) at 24–28 weeks of gestation. The diagnosis of GDM was made when any of the following plasma glucose values were met or exceeded: fasting, 5.1 mmol/L; 1 h, 10.0 mmol/L; 2 h, 8.5 mmol/L.

### Fetal Growth and Neonatal Outcomes

In the follow-up prenatal visits, pregnant women were monitored with routine ultrasound measurements using transabdominal sonography (Philips iU22, Netherlands) to measure fetal head circumference (HC), abdominal circumference (AC), and femur length (FL) to the nearest millimeter in mid pregnancy (18–24 weeks of gestation) and late pregnancy (28–34 weeks of gestation). All ultrasound measurements were conducted by experienced faculty.

Information on fetal sex, date of birth, birth weight (BW), and birth length (BL) was obtained from hospital medical records. Gestational age-adjusted z-scores for fetal biometry, estimated fetal weight (EFW), and newborn BL and BW were constructed according to the INTERGROWTH-21st Standard (20). The INTERGROWTH-21st Standard is a multicenter, multi-ethnic, population-based project, conducted between 2009 and 2014, in eight countries. The project strictly selected eligible pregnancies and assessed longitudinal fetal growth and newborn size to construct prescriptive intrauterine growth standards for each gestational age (21).

Preterm birth was defined as birth at <37 weeks of gestation (20). Small-for-gestational age (SGA) was defined as BW <10th percentile gestational age- and sex-specified BW, and large-for-gestational age (LGA) was defined as BW >90th percentile gestational age- and sex-specified BW based on the INTERGROWTH-21st Standard (21).

### Statistical Analysis

In this analysis, we investigated the relationship between the first trimester FPG concentrations and offspring growth patterns (mid pregnancy, late pregnancy, and at birth) using unbalanced repeated measurement regression models. Since body length cannot be estimated by ultrasound, we used FL instead to assess overall length growth (22). This regression technique considers the correlation of repeated measurements within one subject into account, assesses both the time-independent and time-dependent effects of FPG in early pregnancy, and allows for incomplete data (23). We included early-pregnancy FPG in these models as an intercept and as an interaction term with gestational age to estimate fetal growth rates over time (23). The models performed with R can be written as: fit<-lmer (weight ~ FPG + time + FPG * time + β0 + β1 + βj + (1|id), data = newdata). The term “weight,” including the estimated fetal weight in the second and third trimesters and weight at birth, reflects the time-dependent outcome variable, while “time” is a continuous variable that reflects the gestational week of estimated weight in the second and third trimesters and weight at birth. “FPG” and “FPG * time” reflect the time-independent and time-dependent growth differences, respectively. “(1|id)” refers to a random intercept for subjects (repeated subject = id). “β0 + β1 + βj” is the regression coefficient for covariates 0 to j, where j is the number of covariates. A similar model was used for length growth. Crude models were adjusted for model 1 (including maternal age, parity, education levels, family history of diabetes, and fetal sex) and model 2 (model 1 plus pre-pregnancy BMI).

Differences in fetal weight growth through pregnancy by early GDM and pre-pregnancy BMI were examined by linear mixed modeling. Analysis was repeated with the exception of women with GDM.

Furthermore, we examined the association of early FPG concentrations with fetal growth characteristics in the second and third trimesters and at birth using linear regression models. The results are expressed as *β* coefficients (95% CIs). Crude models were adjusted for model 1 (including maternal age, parity, education levels, family history of diabetes, and fetal sex) and model 2 (model 1 plus pre-pregnancy BMI). Subsequently, we assessed the associations with the risks of adverse birth outcomes using logistic regression models adjusted for the same covariates.

Stratified analyses by maternal characteristics and fetal sex mentioned in the covariates were conducted in each pregnancy period to examine which group was more affected by FPG. Analyses were adjusted for covariates when they were not the strata variables conducted in model 1 and model 2.

To validate the confidence of the association of higher maternal FPG with fetal growth during pregnancy, sensitivity analyses were performed in two steps. First, we repeated the analyses limited to fetuses from mothers without GDM because we were interested in non-diabetic women. The second analysis conducted excluded pregnancies with complications, such as gestational hypertension, preeclampsia, placenta previa, placental abruption, and cholestasis of pregnancy.

Normally distributed variables are presented as the means ± SD; non-normally distributed variables are presented as medians with 95% ranges. All statistical analyses were performed using R statistical software version (package rms, lme4, ggplot).

## Results

### Population Characteristics

Descriptive characteristics of mothers and newborns are listed in **Table 1**. The mean (SD) maternal age was 30.89 (3.86) years, and the mean BMI (SD) was 21.13 (2.71) kg/m^2^. Delivery took place at a median of 39.1 weeks (95% CI: 35.6, 41), and the mean (SD) birth weight was 3,327.67 (437.23) g. A total of 69.22% of women were nulliparous, and 12.95% were overweight/obese. The mean maternal FPG values was 4.49±0.36 mmol/L, with 2,015 (5.6%) women having over 5.1 mmol/L. The rates of LGA, SGA, and preterm birth were 13.13%, 3.29%, and 5.25%, respectively. When compared to women delivering average-for-gestational age (AGA) newborns, higher mean FPG concentrations in early pregnancy were observed in women delivering LGA newborns (4.55±0.38 *vs*. 4.48±0.36 mmol/L, p < 0.001), and lower mean FPG concentrations were observed in mothers who gave birth to babies with SGA (4.45±0.37 *vs*. 4.49 ± 0.36 mmol/L, p = 0.001). Women who gave birth to premature babies also had higher levels of FPG than women who gave birth to term (4.51 ± 0.39 *vs*. 4.49 ± 0.36 mmol/L, p = 0.03).

**Table 1:** Baseline characteristics of study participants.

Characteristics	All (N = 35,981)
Maternal characteristics	
Age, mean±SD, years	30.89 ± 3.86
<35, n (%)	29,387 (81.67)
≥35, n (%)	6,594 (18.33)
BMI, mean±SD, kg/m^2^	21.13 ± 2.71
<18.5, n (%)	4,694 (13.05)
18.5–23.9, n (%)	26,626 (74)
≥24, n (%)	4,661 (12.95)
Nullipara, n (%)	24,905 (69.22)
Family history of diabetes, n (%)	2,491 (6.92)
Maternal education levels, n (%)	
Primary education	6,240 (17.34)
Bachelor’s	24,049 (66.84)
Master’s	5,242 (14.57)
Doctoral	450 (1.25)
FPG in first trimester, mean ±SD, mmol/L	4.49 ± 0.36
FPG ≥5.1 mmol/L, n (%)	2,015 (5.6%)
**Neonatal characteristics**	
Fetal gender (boys, %)	18,520 (51.47)
Gestational weeks, median (95% CI)	39.1 (35.6, 41)
Birth weight, g	3,327.67 ± 437.23
Birth length, mm	49.81 ± 1.41
**Pregnancy complications and outcomes, n (%)**	
GDM	4,758 (13.22)
Preeclampsia	845 (2.35)
Pregnancy-induced hypertension	990 (2.75)
Intrahepatic cholestasis	238 (0.66)
Placental abruption	86 (0.24)
Placenta previa	398 (1.11)
Preterm birth	1,888 (5.25)
LGA	4,723 (13.13)
SGA	1,186 (3.29)

Values are means±SD.

BMI, body mass index; FPG, fasting plasma glucose; GDM, gestational diabetes mellitus; LGA, large-for-gestational age; SGA, small-for-gestational age.

### First-Trimester Fasting Plasma Glucose Concentrations With Fetal Growth and Birth Outcomes

**Figure 2:**
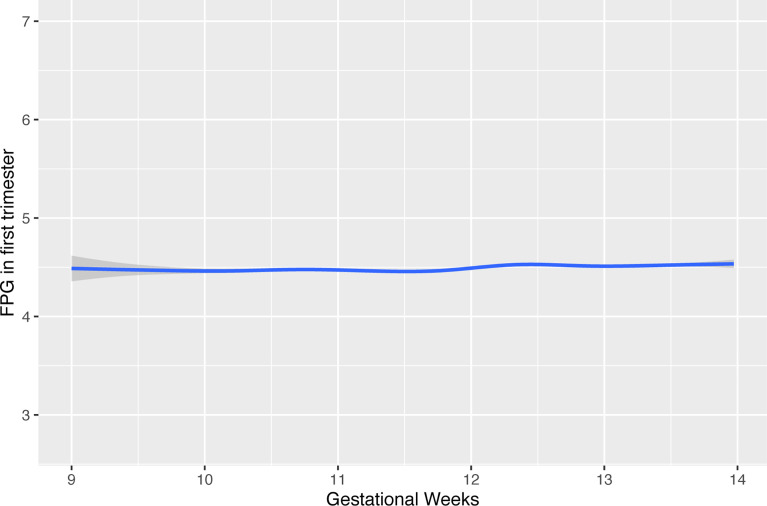
The effect of timing in gestation for FPG values during early pregnancy. FPG, fasting plasma glucose.

Considering the variation in maternal FPG during early pregnancy, we first modeled a curve to evaluate the potential effect of gestational week on maternal FPG values by using a Locally Weighted Scatterplot Smoothing procedure. The results showed a small fluctuation at the end of the first trimester (**Figure 2**). Repeated measurement analysis showed that first-trimester FPG levels were positively associated with the fetal growth trajectory based on fetal weight and length from visits 2 and 3 and delivery [adjusted for model 1: length: 0.046 SD (0.030–0.082), p < 0.001; weight: 0.039 SD (0.014–0.064), p = 0.002]. After additional adjustment for pre-pregnancy BMI, the significant association for fetal weight no longer reached statistical significance.

**Figure 3:**
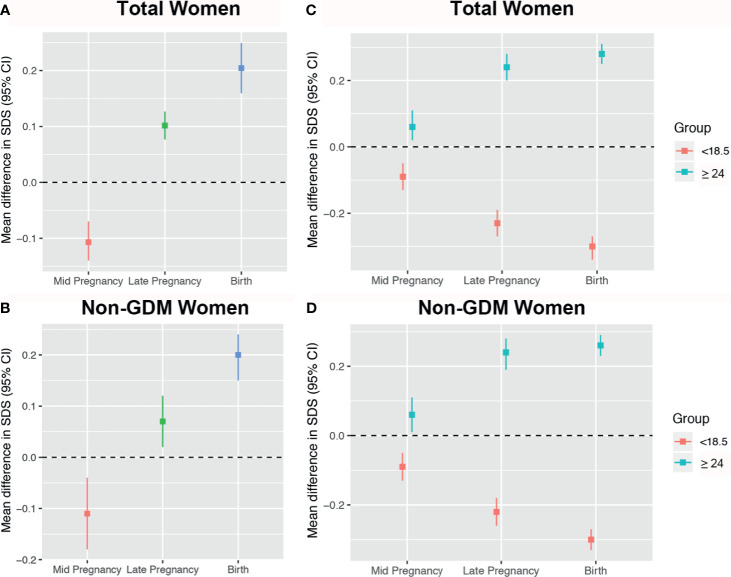
Mean differences in estimated fetal weight (EFW) across pregnancy stratified by early gestational diabetes mellitus (GDM) and pre-pregnancy body mass index (BMI). Women with non-early GDM are the reference group in panels **(A, B)**, represented by the black zero-line. Normal weight women are the reference group in panels **(C, D)**.

In mid pregnancy, fetuses of early GDM mothers had lighter EFW [mean difference in EFW SDS: -0.10 (-0.14, -0.07)] compared to non-early GDM mothers (reference). From this time until birth, they grew faster [difference in mean EFW at late pregnancy and weight at birth was 0.10 (0.07, 0.13) and 0.20 (0.16, 0.25), respectively] (**Figure 3A**). This pattern was maintained after excluding fetuses of mothers who were later diagnosed with GDM (**Figure 3B**). Compared to fetuses of normal-weight women (reference), fetuses of underweight women were smaller from mid to birth [mean difference in EFW SDS: -0.09 (-0.13, -0.05); -0.22 (-0.26, -0.18); -0.3 (-0.33, -0.27), respectively], and fetuses of overweight/obese women were heavier across pregnancy [mean difference in EFW SDS: 0.06 (0.01, 0.11); 0.24 (0.19, 0.28); 0.26 (0.23, 0.29), respectively] (**Figure 3C**). Results were similar after excluding fetuses of mothers who were later diagnosed with GDM (**Figure 3D**).

The effects of maternal first-trimester FPG on fetal growth patterns during each pregnancy period are summarized in **Table 2**. Higher FPG was associated with a pattern of reduction in z-scores for all fetal growth parameters in mid pregnancy in multivariable-adjusted model 2, except for FL. However, as in late pregnancy, higher FPG was associated with increased AC, EFW, and FL SDS in model 1, resulting in significantly increased weight and length at birth. The positive associations between FPG and AC or EFW in late pregnancy lost statistical significance after additional correction for pre-pregnancy BMI, while the association with birth weight and length remained significant in the fully adjusted model.

**Table 2:** Association of maternal early-pregnancy glucose concentrations with fetal growth during pregnancy and birth outcomes.

Period	Basic model	Model 1^†^	Model 2^‡^
	beta (95% CI)	beta (95% CI)	beta (95% CI)
**Mid pregnancy**			
**AC (N = 35,569)**	-0.011 (-0.041, 0.019)	-0.03 (-0.046, 0)^*^	-0.051 (-0.081, -0.02)^*^
**HC (N = 35,532)**	-0.068 (-0.099, -0.037)^*^	-0.074 (-0.105, -0.044)^*^	-0.08 (-0.111, -0.049)^*^
**EFW (N = 35,516)**	-0.037 (-0.069, -0.005)^*^	-0.051 (-0.086, -0.016)^*^	-0.073 (-0.109, -0.037)^*^
**FL (N = 35,698)**	0.032 (0.004, 0.06)^*^	0.022 (-0.007, 0.05)	0.008 (-0.021, 0.037)
**Late pregnancy**			
**AC (N = 35,273)**	0.111 (0.08, 0.142)^*^	0.079 (0.047, 0.111)^*^	0.019 (-0.013, 0.051)
**HC (N = 34,272)**	0.029 (-0.009, 0.067)	0.014 (-0.024, 0.051)	-0.013 (-0.051, 0.025)
**EFW (N =,34,254)**	0.104 (0.073, 0.136)^*^	0.072 (0.04, 0.103)^*^	0.014 (-0.018, 0.046)
**FL (N = 35,261)**	0.071 (0.041, 0.101)^*^	0.059 (0.029, 0.09)^*^	0.048 (0.017, 0.078)^*^
**Birth size**			
**Weight**	0.2 (0.167, 0.223)^*^	0.162 (0.135, 0.189)^*^	0.083 (0.059, 0.066)^*^
**Length**	0.083 (0.062, 0.103)^*^	0.063 (0.042, 0.083)^*^	0.028 (0.007, 0.048)^*^
**Birth outcomes**	**Basic model**	**Model 1^†^**	**Model 2^‡^**
	**OR (95% CI)**	**OR (95% CI)**	**OR (95% CI)**
**LGA**	1.658 (1.528, 1.8)^*^	1.546 (1.422, 1.679)^*^	1.25 (1.148, 1.362)^*^
**SGA**	0.766 (0.652, 0.898)^*^	0.82 (0.697, 0.965)	0.92 (0.781, 1.091)
**Preterm birth**	1.159 (1.022, 1.313)^*^	1.094 (0.963, 1.241)	1.061 (0.932, 1.208)

^†^Adjusted for maternal age, education levels, parity, family history of diabetes, fetal gender, and gestational age of sample collection.

^‡^Adjusted for maternal age, education levels, parity, gestational age of sample collection, pre-pregnancy BMI.

BMI, body mass index; FPG, fasting plasma glucose; GDM, gestational diabetes mellitus; LGA, large-for-gestational age; SGA, small-for-gestational age; AC, abdominal circumference; HC, head circumference; EFW, estimated fetal weight; FL, femur length; OR, odds ratio.

*p < 0.05.

Higher first-trimester FPG was independently associated with an increased risk of LGA after correction for model 2 [odds ratio (OR): 1.250 (95% CI: 1.148, 1.436)]. There was a protective effect on SGA and an increased risk of preterm birth in the crude model [OR_SGA_: 0.766 (95% CI: 0.652, 0.898) and OR_PTB_: 1.159 (95% CI: 1.022, 1.313); respectively], but both became non-significant after adjustments for model 1 and model 2 (**Table 2**).

### Response of Trimester-Specific Fetal Biometry Parameters to First-Trimester Fasting Plasma Glucose Stratified by Known Maternal Phenotypes

**Figure 4:**
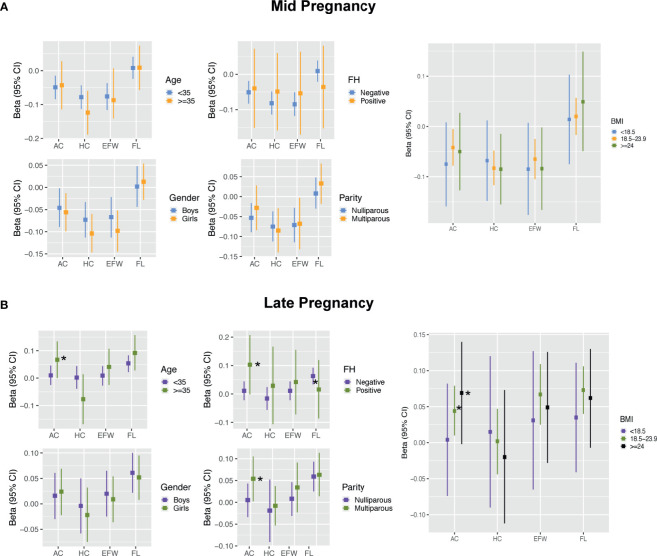
Results for effect modification by maternal and fetal characteristics in mid pregnancy **(A)** and late pregnancy **(B)**. Covariates of adjustment in models: maternal age, parity, pre-pregnancy BMI, gestational age of sample collection, family history of diabetes, and fetal gender. AC, abdominal circumference; HC, head circumference; EFW, estimated fetal weight; FL, femur length; FH, family history of diabetes; BMI, body mass index. *Represent significant association after full adjustment in subgroups for AC.

No interaction terms for maternal traits were found in mid pregnancy (all p-interaction >0.05; **Figure 4A** and **Supplementary Table 2**). In late pregnancy, although no evidence of an association between FPG and AC growth was present in the fully adjusted model, analyses stratified by maternal characteristics showed significantly positive estimates among mothers who were older (35 years), had a family history of diabetes, and had multiparity after controlling for BMI [0.067 SD (0, 0.134), 0.103 SD (-0.001, 0.207), and 0.054 SD (0.002, 0.105), respectively). However, the effect estimates in subjects with age younger than 35 years, without a family history of diabetes, and nulliparity were all close to zero (**Figure 4B** and **Supplementary Table 2**). Analysis stratified by BMI showed that the positive association between FPG and AC was evident in women with BMI ≥18.5 kg/m^2^ (**Figure 4B** and **Supplementary Table 2**). No clearly different effects between groups stratified by age, family history, parity, and BMI were present for FL.

**Figure 5:**
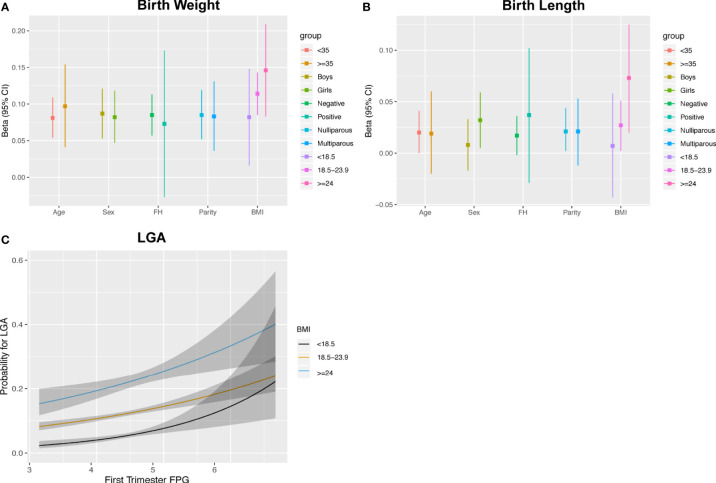
Associations between maternal FPG with birth weight **(A)**, birth length **(B)** and the estimated probability for LGA stratified by pre-pregnancy BMI **(C)**.

The estimates of FPG with birth weight and length were similar between subgroups, except for women carrying female fetuses and those with BMI ≥18.5 kg/m^2^, who showed a pattern of higher estimates for the association between FPG and BL (**Figures 5A, B** and **Supplementary Table 3**). The associations with LGA were similar to the results shown for the continuous birth weight results (**Supplementary Table 3**). However, the curves for LGA showed an incremental separation of their probabilities in the three BMI subgroups with increasing FPG at the lower levels of FPG and exhibited overlap or reversal of their relative probabilities at higher levels (**Figure 5C**). Stratified analyses showed that the OR values (95% CI) of LGA for women who were underweight, normal weight, and overweight/obese were 1.802 (1.285, 2.513), 1.351 (1.219, 1.497), and 1.333 (1.129, 2.574), respectively. There were no statistical associations for SGA and PTB in any subgroup (data not shown).

### Sensitivity Analysis Results

In the sensitivity analyses, the negative relationship between maternal FPG and fetal growth in mid pregnancy and the positive relationship in late pregnancy were not materially changed after excluding pregnant women with GDM. Exclusion of individuals with GDM and further exclusion of individuals with pregnancy complications did not materially change the results (data not shown).

## Discussion

In this study, we showed that higher early FPG levels were associated with lower fetal growth in mid pregnancy, and subsequently compensatory increased growth from late pregnancy, resulting in significantly heavier weight and elevated risk of LGA delivery. The whole associations between maternal FPG and detailed measurements of fetal growth parameters in late pregnancy except for FL were fully explained by maternal pre-pregnancy BMI. Furthermore, we showed that advanced age, multiparity, positive family history of diabetes, and higher BMI were associated with an increased AC response to maternal FPG in early pregnancy. Based on pre-pregnancy BMI stratification, FPG was associated with significantly increased risks of LGA in all the three BMI subgroups.

FPG is one of the most commonly used indicators for diabetes, as it reflects beta cell function and generally indicates the secretion of basal insulin (24). This was the first study using fasting plasma samples to investigate fetal growth trajectories during pregnancy. In the current study, per-unit increases in maternal FPG were associated with slowed mid pregnancy fetal growth. This suggests that hyperglycemia affects embryonic development and is a risk factor for fetal growth retardation in the first half of pregnancy. The underlying biological mechanisms may be attributed to a combination of glucose-mediated effects mediated by mitochondrial function, epigenetic modification, and oxidative stress (25–28). Experimental data have indicated that trophoblast cell exposure to hyperglycemia limits migration and invasion, often with dose–response patterns, which further impedes the normal function of the placental villi and interferes with the placentation process (29). These findings suggest that decreased fetal growth may be associated with the worsening of FPG within the respective normal reference range. Alternatively, other studies also demonstrated that hyperglycemia induced upregulation of the C-X3-C motif chemokine ligand 1 (CX3CL1)/C-X3-C motif chemokine receptor 1 (CX3CR1) signaling pathway, which is known to disturb placental perfusion (30, 31). This notion is supported by a study by Stridsklev et al. (32) showing that higher FPG concentrations during early pregnancy positively correlated with the mid pregnancy pulsatility index of the uterine artery. All of these factors triggered a lower chance of efficient glucose flux on the maternal side.

Although the placenta development as a whole is affected by exposure to hyperglycemia, the role of the fetal compensatory response specific to gestational age cannot be ignored. Human fetal glucose availability relies completely on transplacental glucose from mothers (1). The rate of glucose flux to the fetus is controlled by the maternal-to-fetal glucose concentration gradient across the placenta (33). Hyperglycemia-induced apoptosis of non-proliferative syncytiotrophoblast cells leaves incomplete holes in the placenta, which will cause a large flux of glucose into the fetal blood circulation (34). Data have demonstrated that women with higher FPG levels at baseline had a greater risk of developing GDM at approximately 26 weeks, which coincides with the period of elevated maternal endogenous glucose production and reduced insulin sensitivity (35, 36). Stimulation of fetal insulin secretion by maternal hyperglycemia lowered fetal glycemia, which, in turn, increased the maternal–fetal glucose gradient, resulting in rapid fetal growth and excess fat deposition (33). This might be the biological process of accelerated fetal growth in late pregnancy triggered by maternal FPG. In line with these theories, a study among 184 Asian women observed an intrauterine “catch-up” in GDM-exposed fetuses in late pregnancy (32). Furthermore, the exaggerated maternal–fetal glucose gradient by hyperinsulinemic fetuses further attenuates maternal glucose levels, providing an explanation for our study why women who did not develop GDM also share phenotypic characteristics with obesogenic fetopathy. In another study conducted in the UK, Ong et al. (4) reported that FPG levels assessed in the second trimester in non-GDM mothers independently contributed to neonatal macrosomia. However, due to a lack of data about FPG from periconception or early pregnancy, they were unable to explain the potential biological mechanisms. More challenging is whether early exposure to elevated glucose and subsequently accelerated maturation of fetal beta cells will predispose offspring to metabolic diseases in adulthood. Further study in this population is certainly warranted.

We found that the influences of FPG on accelerated AC and EFW in late pregnancy could be explained by pre-pregnancy BMI but that on birth weight could not. However, the discrepancy is not necessarily contradictory. Body composition in neonates includes but is not limited to those measured in our study (i.e., the head, abdomen, and femur), as well as the trunk and limbs. It is likely that FPG may act preferentially in fetal extremities and the thoracic truncus. As stated in another study by Ong et al. (4), elevated maternal glycemia contributed to offspring fat deposition in the arms and the subscapular and suprailiac regions at birth. The positive relationship with femoral growth from late pregnancy in our study may also provide some clues. It has been well documented that fetal adipocyte proliferation occurs primarily in the third trimester and that AC is a good indicator of fetal fat deposition (37). The subgroup analyses showed that a significantly positive association between FPG and AC was robust to adjustment for BMI among individuals with advanced age, multiparity, and positive family history of diabetes. One possible explanation is that maternal conditions with these traditional risk factors might be profoundly involved in fetal programming through modification of oocyte metabolism, predominantly of their mitochondria, leading to increased susceptibility to or aggravated physiological insulin resistance during pregnancy (38, 39). We further stratified continuous BMI values into categories. AC and EFW in late pregnancy reflected changes in FPG concentrations to a lesser extent in lean women than in normal or obese women. This may be attributed to adiponectin, an antidiabetic adipokine that is richly expressed in lean women to regulate glucose metabolism and therefore attenuate fetal growth (1). Similarly, a study conducted in the UK showed that fetuses exposed to GDM mothers combined with obesity showed the greatest AC growth rates at 28 weeks (40). An unexpected observation was that the effects of FPG on the risk of LGA were strongest in underweight women when FPG levels were at high level. This might partly be explained by the fact that more attention might be given to overweight mothers, and interventional management effectively offsets the adverse pregnancy. In contrast, underweight women were regarded as overlooked. Differential DNA methylated regions might be a potential mechanism linking maternal glucose metabolism and offspring outcomes in different BMI subgroups (41).

In this study, we observed positive associations of first-trimester FPG with FL and BL, even after full adjustment. Both FL and BL are predictors of offspring height. However, few studies have investigated the effect of blood glucose on FL, BL, and height, and more studies are needed to replicate our observations.

The interventions of physical activity and diet counseling have not remarkably benefited those diagnosed with GDM at 24–28 weeks’ gestation. In this regard, the IADSPG recommended that early FPG ≥5.1 mmol/L should be considered to define early GDM and supported immediate intervention of maternal glycemic control. To date, whether women with early-onset GDM could benefit from surveillance and management remains controversial due to lack of evidence from large randomized controlled trials (RCTs) (13), and they only observed the outcomes at birth and ignored the process of intrauterine growth. This finding in our study provides potential clinical implications to encourage future large-scale RCTs to pay extra attention to the fetal growth pattern during the management process.

Some limitations of our study should be considered. First, we cannot exclude bias in the selection of the population, since some of the information was based on the medical records and not all participants provided blood samples. It should be noted that self-reported pre-pregnancy weight was applied to calculate BMI, which may cause bias because women tended to underreport their initial weight and it should be used with caution. Second, this study was a hospital-based cohort, and the homogeneous ethnicity of the cohort may increase the internal validity and weaken the generalizability of our findings to other ethnicities. Third, we did not adjust for gestational weight gain (GWG), as fetal growth is a major component of GWG, and additional adjustment of GWG would thus lead to overadjustment. Last, among Caucasians, the Hadlock equation for estimated fetal weight based on FL, AC, and HC has been more widely employed. Although INTERGROWTH-21st has been used less, it is based on a large number of healthy women from eight countries including China. The INTERGROWTH-21st method had strictly defined protocols for conducting of the ultrasound scans, and the statistical analysis was rigorous. Further research is needed to repeat our study with performance of the Hadlock equation.

## Conclusion

In conclusion, the current findings suggest significant glycemia-related fetal growth deviation and an increased risk of LGA infants, highlighting a potentially imperative need for recognition and management in early pregnancy. This is a first step toward emphasizing that the first trimester is a potentially key window of pregnancy for intervention studies. Additional studies including prospective cohorts or RCTs are needed to confirm a feasible strategy to improve fetal growth and birth outcomes.

## Data Availability Statement

The raw data supporting the conclusions of this article will be made available by the authors, without undue reservation.

## Ethics Statement

The studies involving human participants were reviewed and approved by the Ethics Committee of the International Peace Maternity and Child Health Hospital (GKLW 2019-58). The patients/participants provided their written informed consent to participate in this study.

## Author Contributions

FG designed the study, performed the statistical analysis, and wrote the initial manuscript. YL contributed to the study design and revision of the manuscript. ZD and YZ collected and interpreted the data. CZ critically validated the data. JF reviewed, revised, and approved the submitted version. All authors contributed to the article and approved the submitted version.

## Funding

This work was supported by a grant from the National Key Research and Development Program of China (2018YFC1004602).

## Conflict of Interest

The authors declare that the research was conducted in the absence of any commercial or financial relationships that could be construed as a potential conflict of interest.

## Publisher’s Note

All claims expressed in this article are solely those of the authors and do not necessarily represent those of their affiliated organizations, or those of the publisher, the editors and the reviewers. Any product that may be evaluated in this article, or claim that may be made by its manufacturer, is not guaranteed or endorsed by the publisher.
