# Supplementary material for: Observations of the Effects of Maternal Fasting Plasma Glucose Changes in Early Pregnancy on Fetal Growth Profiles and Birth Outcomes
Source: Front Endocrinol (Lausanne). 2021 Aug 19;12:666194. doi: 10.3389/fendo.2021.666194 (PMC8417376; doi:10.3389/fendo.2021.666194)
Supplement: Supplementary file 1 [file Table_1.docx]

**Supplementary Table 1.** Characteristics of the excluded and included subjects

| **Characteristics** | No FPG measurement  N= 6960 | FPG measurement  N= 35981 | *P*-value **^†^** |
| --- | --- | --- | --- |
| **Maternal characteristics** |  |  |  |
| Age, mean$\pm$SD, years | 30.75$\pm$4.31 | 30.89$\pm$3.86 | 0.061 |
| Body mass index, mean$\pm$SD, kg/m^2^ | 21.01$\pm$2.79 | 21.13$\pm$2.71 | 0.082 |
| Nulliparous (%) | 61.26 | 69.22 | 0.001 |
| Higher education (%) | 66.05 | 82.66 | 0.001 |
| Gestational diabetes (%) | 12.84 | 13.22 | 0.075 |
| Preeclampsia (%) | 2.43 | 2.35 | 0.721 |
| Pregnancy induced hypertension (%) | 2.77 | 2.75 | 0.952 |
| **Birth characteristics** |  |  |  |
| Boys, (%) | 52.36 | 51.47 | 0.176 |
| Gestational age at delivery, median (IQR), weeks | 39 (35.4, 41) | 39.1 (35.6,41) | <0.001 |
| Birth weight, mean (SD), g | 3324.35$\pm$426.21 | 3325.63$\pm$387.53 | 0.584 |
| Preterm birth (%) | 5.86 | 5.25 | 0.039 |
| LGA (%) | 13.88 | 13.13 | 0.094 |
| SGA (%) | 3.18 | 3.29 | 0.63 |

Values are observed data and represent means (SD), medians (IQR) or number of subjects (valid %).
**^†^** Differences in subject characteristics between participants with and without glucose measurements available were evaluated using one-way ANOVA tests for continuous variables and chi-square tests for categorical variables.

Abbreviations: IQR: inter quartile range; SD: Standard deviation; LGA: larger-for-gestational age; SGA: small-for-gestational age.

**Supplementary Table 2.** The associations of FPG and fetal parameters classified by maternal and fetal characteristics

| **Mid pregnancy** | | | | | | | | |
| --- | --- | --- | --- | --- | --- | --- | --- | --- |
| **Modification effects** | **AC**  **beta (95% CI)** | ***P* for interaction** | **HC**  **beta (95% CI)** | ***P* for interaction** | **EFW**  **beta (95% CI)** | ***P* for interaction** | **FL**  **beta (95% CI)** | ***P* for interaction** |
| **Age, years** |  | 0.48 |  | 0.955 |  | 0.531 |  | 0.818 |
| <35 | -0.049 (-0.084, -0.015) |  | -0.076 (-0.107, -0.043) |  | -0.076 (-0.116, -0.037) |  | 0.008 (-0.024, 0.041) |  |
| ≥35 | -0.043 (-0.114,0.0281) |  | -0.124 (-0.189, -0.06) |  | -0.087 (-0.141, 0.007) |  | 0.009 (-0.057, 0.074) |  |
| **Family history** |  | 0.849 |  | 0.511 |  | 0.411 |  | 0.413 |
| Positive | -0.04 (-0.152, 0.072) |  | -0.049 (-0.159, 0.06) |  | -0.027 (-0.155, 0.101) |  | -0.036 (-0.153, 0.081) |  |
| Negative | -0.051 (-0.083, -0.019) |  | -0.082 (-0.114, -0.049) |  | -0.075 (-0.112, -0.038) |  | 0.009 (-0.021, 0.039) |  |
| **Fetal gender** |  | 0.667 |  | 0.156 |  | 0.377 |  | 0.296 |
| Boys | -0.046 (-0.089, -0.002) |  | -0.073 (-0.113, -0.033) |  | -0.067 (-0.113, -0.022) |  | 0.002 (-0.044, 0.048) |  |
| Girls | -0.056 (-0.099, -0.013) |  | -0.104 (-0.147, -0.06) |  | -0.098 (-0.145, -0.052) |  | 0.013 (-0.028, 0.054) |  |
| **Parity** |  | 0.658 |  | 0.883 |  | 0.756 |  | 0.284 |
| Nulliparous | -0.06 (-0.097, -0.023) |  | -0.075 (-0.112, -0.037) |  | -0.071 (-0.114, -0.028) |  | 0.008 (-0.030, 0.048) |  |
| Multiparous | -0.028 (-0.084,0.028) |  | -0.085 (-0.14, -0.029) |  | -0.068 (-0.132, -0.003) |  | 0.033 (-0.018, 0.083) |  |
| **BMI, kg/m^2^** |  | 0.656 |  | 0.097 |  | 0.735 |  | 0.895 |
| <18.5 | -0.075 (-0.159, 0.008) |  | -0.068 (-0.148, 0.012) |  | -0.085 (-0.176, 0.007) |  | 0.014 (-0.075, 0.103) |  |
| 18.5-23.9 | -0.042 (-0.078, -0.005) |  | -0.083 (-0.117, -0.048) |  | -0.065 (-0.105, -0.025) |  | 0.02 (-0.017, 0.057) |  |
| ≥24 | -0.048 (-0.127, 0.027) |  | -0.085 (-0.155, -0.015) |  | -0.084 (-0.166, -0.002) |  | 0.049 (-0.049, 0.149) |  |
| **Late pregnancy** | | | | | | | | |
| **Age, years** |  | 0.327 |  | 0.172 |  | 0.455 |  | 0.367 |
| <35 | 0.01(-0.025, 0.046) |  | 0.002 (-0.039, 0.044) |  | 0.009 (-0.027, 0.044) |  | 0.054 (0.022, 0.083) |  |
| ≥35 | 0.067 (0, 0.134) |  | -0.077 (-0.168, 0.014) |  | 0.041 (-0.025, 0.107) |  | 0.092 0.028, 0.157) |  |
| **Family history** |  | 0.109 |  | 0.369 |  | 0.603 |  | 0.25 |
| Positive | 0.103 (-0.001, 0.207) |  | 0.029 (-0.106, 0.166) |  | 0.042 (-0.072, 0.155) |  | 0.016 (-0.086, 0.119) |  |
| Negative | 0.011 (-0.022, 0.044) |  | -0.016 (-0.056, 0.024) |  | 0.011 (-0.022, 0.044) |  | 0.063 (0.034, 0.092) |  |
| **Fetal gender** |  | 0.8 |  | 0.306 |  | 0.241 |  | 0.996 |
| Boys | 0.016 (-0.030, 0.061) |  | -0.004 (-0.058, 0.05) |  | 0.02 (-0.025, 0.065) |  | 0.061 (0.022, 0.1) |  |
| Girls | 0.024 (-0.022, 0.069) |  | -0.022 (-0.075, 0.032) |  | 0.009 (-0.036, 0.054) |  | 0.052 (0.008, 0.095) |  |
| **Parity** |  | 0.137 |  | 0.677 |  | 0.358 |  | 0.529 |
| Nulliparous | 0.005 (-0.034, 0.043) |  | -0.019 (-0.091, 0.052) |  | 0.008 (-0.031, 0.046) |  | 0.059 (0.025, 0.093) |  |
| Multiparous | 0.054 (0.002, 0.105) |  | -0.008 (-0.053, 0.037) |  | 0.034 (-0.023, 0.091) |  | 0.063 (0.014, 0.113) |  |
| **BMI, kg/m^2^** |  | 0.518 |  | 0.371 |  | 0.089 |  | 0.932 |
| <18.5 | 0.004 (-0.074, 0.082) |  | 0.015 (-0.09,0.12) |  | 0.031 (-0.065, 0.127) |  | 0.035 (-0.041,0.111) |  |
| 18.5-23.9 | 0.044 (0.01, 0.079) |  | 0.002 (-0.044, 0.047) |  | 0.067 (0.025, 0.109) |  | 0.070 (0.040, 0.106) |  |
| ≥24 | 0.069 (-0.002, 0.14) |  | -0.02 (-0.112, 0.073) |  | 0.049 (-0.028, 0.126) |  | 0.062 (-0.007, 0.13) |  |

Covariates of adjustment in models: maternal age, parity, pre-pregnancy BMI, gestational age of sample collection, family history of diabetes and fetal gender.

Abbreviation: AC: Abdominal circumference; HC: head circumference; EFW: Estimated fetal weight; FL: Femur length; BMI: Body mass index.

**Supplementary Table 3**. The associations of fasting plasma glucose and birth size classified by maternal and fetal characteristics

| Modification effects | Birth weight | | *P* for interaction | Birth length | *P* for | | | LGA *P* for | | | | |  |  |
| --- | --- | --- | --- | --- | --- | --- | --- | --- | --- | --- | --- | --- | --- | --- |
|  | Beta (95% CI) | |  | Beta (95% CI) | | interaction | | OR (95% CI) interaction | | | | |  |  |
| Age, years |  | | 0.63 |  | | 0.428 | | 0.319 | | | | |  |  |
| <35 | 0.081 (0.054, 0.109) | |  | 0.02 (0, 0.041) | |  | | 1.207 (1.094, 1.331) | | | | |  |  |
| ≥35 | 0.097 (0.041, 0.154) | |  | 0.019 (-0.02, 0.06) | |  | | 1.404 (1.178, 1.673) | | | | |  |  |
| Family history | |  | 0.075 |  | | | 0.97 | | 0.027 |  | |  |  |  |
| Positive | 0.073 (-0.027, 0.173) | |  | 0.037 (-0.029, 0.102) | |  | | 1.044 (0.784, 1.386) | | | | |  |  |
| Negative | 0.085 (0.057,0.113) | |  | 0.017 (-0.002, 0.036) | |  | | 1.278 (1.168,1.398) | | | | |  |  |
| Fetal gender |  |  | 0.327 |  | | | 0.063 | | 0.235 |  | |  |  |  |
| Boy | 0.087 (0.053, 0.121) | |  | 0.008 (-0.017, 0.033) | |  | | 1.296 (1.147, 1.462) | | | | |  |  |
| Girl | 0.082 (0.047, 0.118) | |  | 0.032 (0.005, 0.059) | |  | | 1.21 (1.073, 1.364) | | | | |  |  |
| Parity |  |  | 0.687 |  | | | 0.87 | | 0.653 |  | |  |  |  |
| Nulliparous | 0.085 (0.052, 0.119) | |  | 0.021 (-0.002, 0.044) | |  | | 1.229 (1.101, 1.372) | | |  |  |  |  |
| Multiparous | 0.083 (0.036, 0.131) | |  | 0.021 (-0.012, 0.053) | |  | | 1.29 (1.132, 1.485) | | | | | |  |
| BMI, kg/m^2^ |  |  | 0.128 |  | | | 0.39 | | 0.001 |  |  |  |  |  |
| <18.5 | 0.082 (0.016, 0.148) | |  | 0.007 (-0.043, 0.058) | |  | | 1.802 (1.285, 2.513) | | | | | | |
| 18.5-23.9 | 0.114 (0.085, 0.143) | |  | 0.027 (0.002, 0.051) | |  | | 1.351 (1.219, 1.497) | | |  |  |  |  |
| ≥24 | 0.146 (0.083, 0.209) | |  | 0.073 (0.02, 0.125) | |  | | 1.333 (1.129, 1.574) | | |  |  |  |  |

Covariates of adjustment in models: maternal age, parity, pre-pregnancy BMI, gestational age of sample collection, family history of diabetes and fetal gender.

Abbreviations: LGA: larger-for-gestational age; BMI: body mass index; OR: odds ratio.
